# Hepatitis C Virus Infection and Mixed Cryoglobulinemia

**DOI:** 10.1155/2012/502156

**Published:** 2012-07-10

**Authors:** Gianfranco Lauletta, Sabino Russi, Vincenza Conteduca, Loredana Sansonno

**Affiliations:** ^1^Department of Biomedical Sciences and Human Oncology, Section of Internal Medicine and Clinical Oncology, Liver Unit, University of Bari Medical School 70124, Bari, Italy; ^2^Department of Biomedical Sciences, University of Foggia, 71122 Foggia, Italy

## Abstract

Hepatitis C virus (HCV) chronic infection is recognized as the major cause of mixed cryoglobulinemia (MC). Its persistence represents a continuous stimulus for host immune system with production of circulating immune complexes (ICs), one-third of them with cryoprecipitate property. Several factors contribute to the biological activities of ICs, many of which are not completely known. Among them, complement factors play a crucial role in the cold-insoluble ICs-mediated vasculitis, involving primarily small blood vessels in different tissues including skin, kidney, peripheral, and central nervous system. Liver represents the major target of HCV infection with inflammatory infiltrates, resembling secondary lymphoid follicles. Cytokine like CXCL13 contribute to B-cell homing in intraportal lymphoid aggregates, in which B-cell clonal selection may arise. B-cell clonal expansion starts as an antigen-driven event and expands towards indolent and malignant B-cell proliferation. Occurrence of intrahepatic B-cell clonalities correlates with extrahepatic clinical manifestations of HCV infection. In this context, cryoglobulinemic patients should be considered a peculiar HCV-infected population that needs a clinical multidisciplinary approach and more articulated therapeutic measures.

## 1. Introduction

Hepatitis C virus (HCV) is a Flaviviridae family member, genus *Hepacivirus*, infecting about 200 million people worldwide [1]. About 80% of HCV-infected patients develop chronic hepatitis. Among them, 10–20% evolve into cirrhosis, while 1–5% of cirrhotic patients display an hepatocarcinoma [2]. Although HCV is primarily hepatopathic, its clinical feature is characterized by the emergence of several extrahepatic manifestations. Mixed cryoglobulinemia (MC), recognized as the most common HCV-induced extrahepatic disease, is an immune-complex-mediated vasculitis involving small vessels characterized by an underlying B cell proliferation [3]. Since B-cell clonal expansion is hallmark of MC, B-cell malignant evolution may reflect the occurrence of additional genetic accidents [4].

Here, we will discuss the currently accepted pathogenetic mechanisms that characterize cryoglobulinemic vasculitis with its peculiar clinical manifestations, the molecular events proposed to explain the potentially malignant evolution, and the current therapeutic approaches. 

## 2. The Virus

HCV genome is about 9,600 kb length and encodes for a single protein from an open reading frame of over 9024 nucleotides. This single polyprotein is subsequently cleaved into several structural and nonstructural proteins. The structural proteins are represented by core and two envelope proteins (E1 and E2), starting from the 5′ end [1]. The ion channel protein p7 derives from E2 cleavage [5] and is followed by the six nonstructural proteins, namely, NS2, NS3, NS4A, NS4B, NS5A, and NS5B. In addition, another protein called F or ARFP can be produced from a frame-shift of the core protein [6]. At the 5′ and 3′ ends of HCV genome there are two untranslated regions (UTR); the 5′UTR is a highly conserved region constituted by 341 nucleotides that contains an internal ribosome entry site (IRES) for translation. The 3′UTR is constituted by 200 to 235 nucleotides and contains a variable region, a poly U/UC stretch and a highly conserved 98 nucleotide sequence [7].

**Figure 1:**
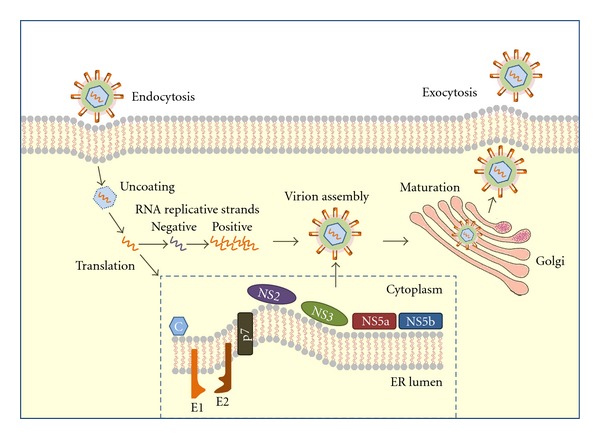
HCV life cycle in host cell. During the replicative stage, after endocytosis, HCV genomic RNA is transcribed into a complementary (negative) RNA strand. After genome amplification and structural and nonstructural viral protein expression, progeny virions are assembled and released.

During the replicative stage, HCV genomic RNA is transcribed into a complementary RNA strand. This “negative” strand constitutes a template for a new genomic synthesis and its identification represents a convincing evidence of active replication [8]. Viral proteins are the result of a co- and post-translational cleavage of a single polyprotein, while host peptidases catalyze the cleavage of structural proteins. HCV particles form a membrane-associated replication complex; after genome amplification and protein expression, progeny virions are assembled and released [9, 10] (Figure 1).

## 3. The Cryoglobulins

Cryoglobulins are immunoglobulins (Igs) characterized by insolubility at low temperature (below 37°C) and redissolving after warming. The first observation of a cryoprecipitation was registered in the serum of a patient affected by multiple myeloma in 1933 [11], even if the term “cryoglobulin” was introduced by Lerner and Watson in 1947 [12]. Meltzer and Franklin first described the cryoglobulinemic syndrome in 29 patients associating cryoglobulin production to a symptomatologic clinical triad characterized by purpura, arthralgias, and weakness [13], with increased serum levels of rheumatoid factor (RF) and/or organ dysfunction. 

On the basis of their immunochemical composition, cryoglobulins are classified as single (type I) or mixed (type II and III) [14]. Type I cryoglobulinemia consists of a monoclonal Ig, more frequently of IgM or IgG isotype. IgM cryoglobulins occurs in almost 6% of malignant IgM paraproteinemias, whereas IgG cryoglobulins characterize almost 2% of all myelomas. Type I IgA cryoglobulins are rare [15]. Type II MC accounts for 50–60% of all cryoglobulins. It comprises an IgM monoclonal component, frequently mounting light k chains, and polyclonal IgG. IgM molecules display a rheumatoid factor activity capable of reacting with intact IgG and/or its F(ab)2′ fragment [16]. No monoclonal component is contained in type III MC that accounts for 30–40% of cryoglobulins. Some authors have noted that type III MC may represent a transition form evolving into type II MC [17]. 

**Figure 2:**
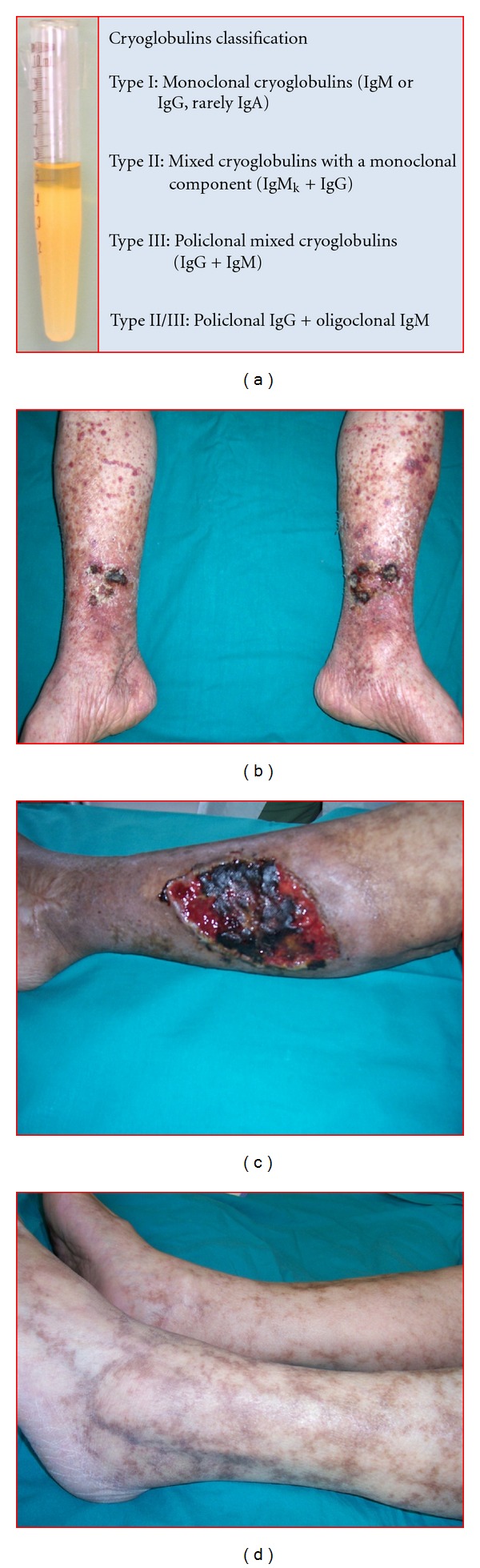
Clinical aspects of cryoglobulinemia. (a) Cryoglobulins classification; (b) lower limbs purpuric manifestations; (c) cutaneous ulcers; (d) livedo reticularis.

Mixed cryoglobulins are potentially present in the course of connective tissue and autoimmune diseases, and chronic infections [18, 19]. The term “essential” defines cryoglobulinemic syndromes without an underlying identifiable disease. It is now accepted that the majority of them occurs in HCV chronically infected patients [20] as the result of specific interactions between the virus and the host immune system [21]. The clinical picture is characterized by the cutaneous manifestations ranging from palpable purpura of lower limbs to chronic torpid cutaneous ulcers more frequent in the supramalleolar regions. Skin reactions include Raynaud's phenomenon, *livedo* *reticularis*, urticarial, and edema (Figure 2). Arthralgias more frequently involve the hands and knees symmetrically. Weakness is nearly always present. Kidney, liver, and nervous system are frequently involved. Renal injury may complicate MC in almost 30% of cases and in 20% of whom nephropathy is present at the diagnosis [22–24]. Clinical features like hypertension, proteinuria, microhematuria, red blood cell casts, and renal failure have an indolent course in about 50% of cases. Less common are nephritic (14%) or nephrotic (21%) syndromes [25]. A defined picture of cryoglobulinemic glomerulonephritis evolve into chronic renal failure in 14% of cases after a mean followup of 6 years [26].

Although kidney involvement is a common feature of systemic vasculitis, cryoglobulinemic nephropathy is considered as a distinct clinical and pathological entity and the etiological role of HCV has been extensively investigated [27]. Type I membranoproliferative glomerulonephritis is predominantly associated with HCV infection [28, 29]. The mechanism of HCV-induced renal damage is unclear. HCV core protein resulted homogeneously distributed along the glomerular capillary wall and tubulo-interstitial blood vessels [30] in association with an anticore activity, suggesting a major role of these immune complexes in the pathogenesis of renal damage [31].

The involvement of the nervous system in the course of HCV-related MC ranges from 17% to 60% [32]. Sometimes, peripheral neuropathy can represent the first clinical sign of cryoglobulinemia [33]. Peripheral nervous system involvement presenting with sensory-motor neuropathy especially of the lower limbs, is often characterized by paresthesias with loss of strength, pain, and burning sensations [34]. Less frequent is central nervous system involvement, characterized by transient dysarthria, hemiplegia, and confusional state [35].

Liver is involved in almost 70% of cases, often with a histopathologic picture of chronic active hepatitis with or without cirrhosis [36, 37]. 

Less common clinical pictures of cryoglobulinemic vasculitis are represented by gastrointestinal (2–6%) and pulmonary (5%) involvement. Intestinal ischaemia may arise with acute abdominal pain; intestinal perforation is also described as well as symptoms that mimic cholecystitis and/or pancreatitis [38]. Interstitial pneumopathy may characterize patients displaying dyspnea and dry cough, whereas an acute alveolar haemorrhage with haemoptysis, respiratory failure, and a radiologic demonstration of multiple infiltrates is rare [39, 40].

## 4. HCV Chronic Infection and MC

After the identification of HCV as the etiologic agent of non-A, non-B chronic hepatitis and the availability of a serologic test for the demonstration of IgG anti-HCV in the early 1990s, several authors described an intriguing association between HCV infection and “essential” MC, apart from some geographical differences [36, 41, 42]. These association was subsequently confirmed by detection of viral genome in sera of cryoglobulinemic patients with a selective concentration in cryoprecipitates [21, 43]. Incidence of HCV infection in MC ranges from 40 to 90% [22]. Otherwise, HCV-negative MC accounts for about 5–10% [44]. 

The intrinsic mechanism by which HCV promotes cryoglobulin production is unclear. Virus persistence, therefore, may represent a continuous stimulus for host immune system unable to produce neutralizing antibodies [45, 46]. In this context, cryoglobulins may represent the product of virus-host interactions in HCV-infected patients, whereas the production of IgM molecules with RF activity is a crucial event in the cryoprecipitating process [22]. The majority of these IgM molecules are almost always associated with light chain cross-idiotype 17.109 and heavy chain cross idiotype G6 [47]. These cross-idiotypes are considered as the product of a restricted expression of germline genes [19].

It has been hypothesized that the composition of ICs in the course of chronic HCV infection includes IgM-17.109 RF molecules which bind anti-HCV IgG [48]. Among viral antigens, the core protein plays a crucial role in cryoglobulins constitution being the relevant ligand for IgG [31]. Interaction between HCV and lymphocytes is capable of modulating cell functions; in particular, an *in* *vivo* activation and expansion of CD5-positive B cells has been considered the major source of IgM RF molecules in type III MC [49, 50]. Therefore, it has been postulated that an initial activation of these cells may be followed by the emergence of a dominant clone that synthesize a monoclonal RF supporting the development of type II MC after a transition phase in which an IgM clonal heterogeneity may define a type II-type III variant [17]. In a subset of HCV-positive patients with MC, a clonal expansion of IgM^+^CD27^+^ B cells expressing hyper-mutated RF-like Ig has been demonstrated in peripheral blood in association to V_H_1–69/J_H_4 and V_H_3–20 gene segment restriction [51]. These findings have been interpreted as a B-cell proliferation induced by specific antigen stimulation, thus sustaining the notion that persistent B-cell stimulation may represent a first step to malignant evolution. 

A crucial role in the composition of cryoprecipitating ICs is played by complement system. Generally, complement binding to setting up ICs decreases the size maintaining them in solution [52]. Mean levels of C3 and C4 fractions in the soluble phase of MC patients' sera correlate to very low amounts in cryoprecipitates thus suggesting the existence of two different compartments characterized by a distinct complement activation [22]. On the contrary, C1q protein and C1q binding activity result significantly enriched in the cryoprecipitates [31]. These data support the hypothesis that an efficient engagement of C1q protein by cryoglobulins may represent a crucial factor in the pathogenetic pathway of MC.

HCV-encoded core protein interacts directly with the receptor for the globular domain of C1q protein (gC1q-R) representing an efficient way to affect the host T- and B-cell immunity. This interaction has been considered capable of modulate T-cell immune response and, on the other hand, circulating HCV core protein engagement with gC1q-R expressed on the surface of B-lymphocytes may represent a direct way by which the virus can affect host immunity [53–55]. The wide expression of gC1q-R on the surface of both circulating blood immunocytes and endothelial cells may determine a specific binding to HCV core protein-containing ICs.

**Figure 3:**
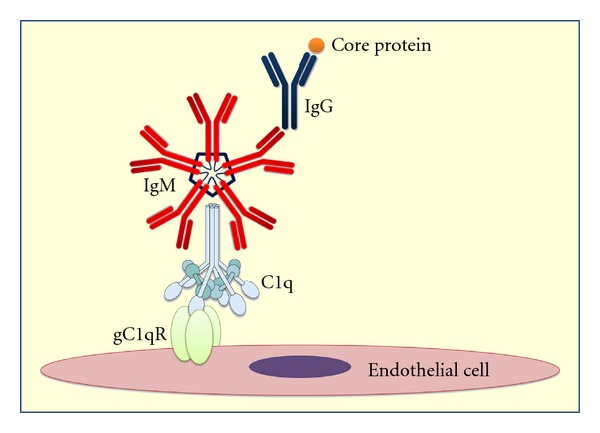
Pathogenetic model of cryoglobulinemic tissue damage. HCV core protein, which has been detected in cryoprecipitate immune complexes, interacts with C1q protein and the receptor for the globular domain of C1q protein (gC1q-R) on the surface of both circulating blood and endothelial cells. Cryoprecipitating immune complexes, including gC1qR complexed to HCV core and C1q proteins, bind in turn IgM molecules with rheumatoid factor activity linked to anti-HCV IgG.

Recently, it has been demonstrated that MC patients display higher levels of soluble gC1q-R that reflects a higher specific mRNA expression in blood mononuclear cells [56]. It was also demonstrated that soluble gC1q-R circulates as a complexed form containing both C1q and HCV core protein in two different binding sites of the molecule (Figure 3). 

C4d, a low-molecular-weight fragment derived from the cleavage of C4 complement fraction following classic complement pathway activation, results are lower in MC patients' sera than in chronic HCV carriers or in healthy subjects [56]. Otherwise, C4d fragment deposits characterize almost all skin biopsy samples of cryoglobulinemic vasculitis. These data lead to hypothesize that low circulating C4d levels are the result of sequestered fragments in the vascular bed. *In* *vitro* experiments showed a peculiar property of MC patients in that, in step with HCV core inhibition of the peripheral blood lymphocytes (PBL) proliferative response, large amounts of soluble gC1q-R were found in culture supernatants. It can be inferred that gC1q-R synthesis and its release from PBL are HCV core mediated and negatively regulated by cell proliferation [56].

In conclusion, in the presence of high levels of circulating gC1q-R, HCV core protein can exacerbate the inflammatory condition by activation of complement cascade thus determining endothelial cell activation starting an *in* *situ* inflammatory response. From a biological point of view, clinical response to antiviral therapy is characterized by a significant reduction of soluble gC1q-R associated to increased levels of C4d and lower viral load [56].

## 5. HCV Infection and Lymphoid Cells

**Figure 4:**
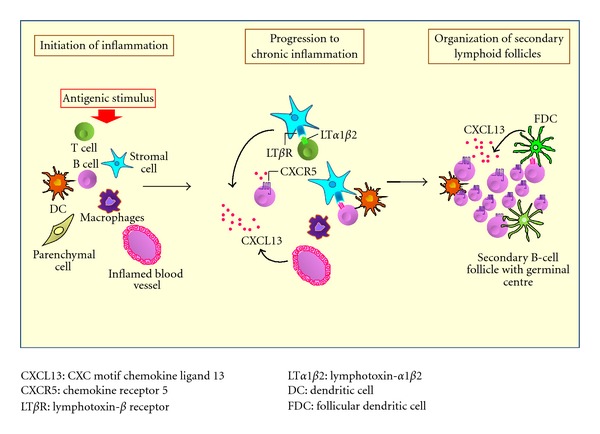
Schematic representation of chronic inflammation and organization of secondary lymphoid follicles in HCV chronic infection. The ability of HCV to chronically persist in the host may represent a continuous stimulus for the immune system resulting in B-cell oligo/monoclonal expansions with selective advantage to clones depending on antigen stimulation. Some chemokines may play a crucial role in the establishment of an adequate microenvironment for activation and expansion of B-lymphocytes in response to signals provided by antigen-presenting cells. Among them, CXC motif chemokine ligand 13 (CXCL13) and its chemokine receptor 5 (CXCR5) are important for secondary lymphoid tissue development and distribution of lymphocytes within microenvironments. CXCL13 is released by endothelial and stromal cells mediated by lymphotoxin-*β* receptor (LT*β*R) signaling and contributes to lymphoid homing in the liver by the creation of a favourable microenvironment sustaining focal B-cell aggregation similar to lymphoid follicles.

HCV is capable of directly modulate B- and T-cells functions [57]. The monoclonal IgM RF production can be considered as the expression of a single dominant clone following the initial stimulation, thus supporting type II MC development [17, 50]. The ability of HCV to chronically persist in the host may represent a continuous stimulus for the immune system resulting in B-cell oligo/monoclonal expansions (Figure 4) [4]. 

HCV recognizes different binding molecules on cells surface that are not completely identified. Among them, the most known are CD81 [58], scavenger receptor class B type I [59], and low-density lipoprotein receptor [60]. MC patients are distinctly characterized by higher levels of cell-associated viral load, because a significant enrichment of HCV RNA in PBL has been demonstrated [61]. This peculiar feature may be considered as the result of a higher density [62] and/or polymorphism of receptor genes [63, 64], whereas direct infection and replication of HCV in B cells may promote lymphocyte proliferation [65]. 

The presence of HCV minus-strand RNA is the key factor to demonstrate an active viral replication in cells, whereas the presence of plus-strand RNA may indicate a possible contamination by circulating virions. By means of a highly specific and sensitive method for the detection of HCV RNA minus strand an active viral replication in lymphoid cells from MC patients has been demonstrated [66]. These results suggest that there is a direct correlation between HCV active infection of lymphoid cells and MC. In a cohort of MC patients, PBL may be considered another HCV productive infection compartment, in addition to the liver, representing a circulating reservoir of HCV infection [67].

Although no specific viral protein has been indicated as BCR ligand [68], analysis of Ig variable gene (IgV) sustain an antigen-driven B-cells expansion. IgV heavy and light chain genes are always mutated as occurs in germinal or post-germinal center origin of B cells [69, 70]. The presence of hypermutated IgV genes capable of recognizing a single epitope suggests that they arise randomly from the B cell pool [70] selected for non-self-antigens. Otherwise, most B-cell expansions show a CDR3 with significant homology to RF-CDR3 [68, 70], suggesting a distinct pathogenesis since these B-cell clonalities derive from precursors with auto-IgG specificity [71]. It has been demonstrated that in some HCV-positive MC patients, BCR recognize IgG-Fc and HCV-NS3 domains, suggesting that its specificity derives from a cross-reactivity between a virus-associated epitope and IgG autoantigen [72]. This mechanism may also contribute to the virus enrichment on the lymphoid cells in MC patients, thus conditioning RF B cells to undergo cell cycle and secrete RF molecules [73]. 

## 6. HCV and Lymphoproliferation

In the course of B-cell proliferation, several mutants may derive from IgV genes somatic mutations. By means of polymerase chain reaction technique (PCR) directed against the variable-determining-joining region (VDJ), it is possible to identify the combination of N regions along with different DH and JH regions. This unique combination represents a clonal marker of cell progeny. The application of this method leads to the demonstration that B-cell clonal expansions are present in the liver tissue of almost 90% of HCV-positive MC patients if compared with blood and bone-marrow compartments [73].

HCV chronic infection is characterized by the development of inflammatory infiltrates involving the portal tracts. These infiltrates often appear as follicle-like structures resembling a germinal center functionally active [74, 75]. VDJ pattern obtained from these patients resulted in oligoclonality to monoclonality, suggesting that intrahepatic B-cell expansions raise from very few or single cells. In addition, each focus may derive from different B cell of the polyclonal repertoire, resulting in the development of unrelated clones.

The occurrence of intrahepatic B-cell clonal expansions profoundly influenced the clinical spectrum of HCV infection, since it was associated invariably with extrahepatic manifestations including cryoglobulinemia, high serum levels of RF activity, monoclonal gammopathy of undetermined significance, and also B-cell non Hodgkin lymphoma. Clonal expansions display a restricted V gene usage, thus confirming a direct relation with clinical manifestations [76]. In addition, sequence analyses of IgH CDR3 gene segments of intraportal B-cell clonalities revealed a wide range of variations, suggesting that they are the result of an antigen-driven response [77]. These findings lead to hypothesize that B-cell clones start expanding in the liver as a consequence of an upregulated IgH-VDJ mutational activity and then migrates in the circle and also bone marrow [76].

However, the relationship between emergence and persistence of intrahepatic or circulating B-cell clones and HCV infection remains unclear. Several pieces of evidence indicate that some chemokines can play a crucial role in the establishment of an adequate microenvironment for activation and expansion of B-lymphocytes in response to signals provided by antigen-presenting cells [78]. Among them, CXC ligand 13 (CXCL13), also known as B-cell attracting chemokine 1 or B-lymphocyte chemoattractant, is important for secondary lymphoid tissue development and distribution of lymphocytes within microenvironments [79]. High serum levels of CXCL13 protein in MC patients paralleled those of specific mRNA expression in liver and skin tissues, suggesting that this chemokine may represent a key factor in the pathogenesis of cryoglobulin-induced damage [80]. CXCL13 contributes therefore to lymphoid homing in the liver by creating a local microenvironment sustaining focal B-cell aggregation similar to lymphoid follicles (Figure 3). 

In addition, B-cell enrichment may be the result of the presence of some signals enhancing cell survival [81]. B-lymphocyte activating factor (BAFF), also known as B-lymphocyte stimulator (BLyS), is expressed and secreted by activated monocytes, macrophages, and dendritic cells. Serum BAFF levels results increased in patients with chronic HCV infection, as well as in other autoimmune diseases like systemic lupus erythematosus and rheumatoid arthritis, and this was correlated to autoimmune and vasculitic manifestations. The increased levels of BAFF may modulate the sensitivity of B cells to apoptosis prolonging their survival, thus representing another possible factor in the clonal B-cell expansion [82].

## 7. Management of MC

The main goals of the therapy of MC are represented by: (a) eradication of HCV infection; (b) deletion of the underlying B-cell clonal expansions; (c) depletion of cryoproteins. 

Conventionally, in the pre-HCV era, management of MC was based on the use of corticosteroids and immunosuppressive drugs. Following the empirical observation in 1987 of the effectiveness of recombinant IFN-*α* in 7 patients with “essential” MC [83], and the subsequent demonstration of the pathogenetic role of HCV [21], IFN-*α* became a rational therapeutic strategy. The introduction of pegylated IFN-*α* changed the therapeutic scenario of chronic hepatitis C increasing virological responses [84, 85] as well as the introduction of ribavirin (RBV), a nucleoside antimetabolite agent [86]. This combination, now considered the standard of care (SoC) for HCV management [87], has been shown to be effective in a remarkable proportion of HCV-related MC patients, resulting in a complete clinical response and sustained virological response (SVR) in 78% of the patients [88]. In addition, serum levels of C3 and C4 complement fractions normalized in 80% and cryoglobulins disappeared in 56% of the patients. Even when the antiviral treatment results in resolution of vasculitis, no or only partial improvement in neuropathy and glomerulonephritis is observed, suggesting that the clinical outcome may be conditioned by factors other than the virus [22].

It should also be emphasized that the occurrence of B-cell clonal expansions is able to influence the clinical expression of HCV infection, in that it is consistently associated with extrahepatic manifestations, like MC [76, 89, 90]. Enrichment of B-cell clones in at least three different compartments, namely, liver, bone marrow, and the circulation, and expansion of RF-synthesizing B cells are the biological hallmark of MC [22]. Consequently, deletion of B-cell clonalities may provide a rational way to treat MC. It is well known that CD20 antigen, a transmembrane protein, is selectively expressed on pre-B and mature lymphocytes, and that CD20-positive cells are remarkably expanded and activated in patients with MC [61, 91]. 

Since rituximab (RTX), a chimeric moAb specifically directed to CD20 antigen, has been shown to be therapeutically effective in autoimmune and lymphoproliferative disorders [92–94], it seemed logical to propose its use in HCV-related MC patients refractory to, or relapsing after, conventional antiviral therapy. The first papers about the use of RTX in HCV-related MC [95, 96] showed that it is an effective, safe, and well-tolerated treatment for type II MC patients, including those resistant to, or frequently recurring after, previous treatments. However, a not negligible drawback is the frequently increased viremia in the responders. On these bases, several subsequent papers have addressed the issue of the use of RTX, alone or in combination with steroids [97, 98]

In our own study [99], a triple therapeutic combination (pIFN-*α* plus RBV plus RTX), designated with the acronym PIRR, was administered to 22 HCV-positive MC patients, whereas 15 additional patients with the same pathology received, by comparison, pIFN-*α* plus RBV with the exclusion of RTX. Followup was protracted for 36 months from the end of treatment. Results showed a complete response in 54.5% of patients treated with PIRR, and only in 33.3% of those who were given pIFN-*α* plus RBV without RTX (*P* < 0.05). Even more interesting were the observations that: (a) in the large majority (83.3%) of the responders belonging to the PIRR-treated group, a conversion of B-cell populations from oligoclonal to polyclonal was recorded in the liver, bone marrow, and peripheral blood compartments; (b) compared with 40% of the control group, in all patients of the PIRR group the CR was maintained throughout the follow-up period. Whether RTX should be administered to patients with cryoglobulinemic vasculitis as first- or second-line therapy remains to be established [100]. 

Of particular interest is the question about MC patients that do not obtain an SVR or those patients showing a continuous cryoglobulin production despite virus eradication. In the first case the use of the new direct-acting antivirals (DAAs) like Telaprevir or Boceprevir (recently approved by the FDA for the treatment of HCV genotype 1 chronic infection) may represent a further therapeutic option [101]. Persistence of MC vasculitis in patients achieving a SVR represents an emerging picture following antiviral and B-cell depletive combined therapies [102, 103]. In these patients a different immunochemical structure of circulating immune-complexes may be postulated; the use of corticosteroids, cyclophosphamide, RTX, or ofatumumab (an IgG1k fully humanized CD20 MoAb) may be considered [104].

Therapeutic apheresis is a palliative procedure that can be extremely useful for the treatment of severe, life-threatening vasculitis [100] as well as for the treatment of chronic leg ulcers in patients resistant to other therapies [105].

Others additional therapeutic approaches for MC have been proposed, like tyrosine kinase inhibitor imatinib, antiangiogenic drugs like thalidomide, bortezomib (a proteasome inhibitor), and IL-2, but future controlled studies are required to establish if these agents will improve MC therapy [106, 107].

## 8. Conclusions

Although the major role of HCV in the production of cryoglobulins and systemic vasculitis has been clearly established, there are several aspects in the pathogenesis of MC that still require further investigations. Particularly interesting is the B-cell expansion process that starts as a consequence of viral persistence, with preferential involvement of RF-producing B cells. This process seems to occur in a microenvironment like intraportal lymphoid follicles as a result of a distinct selection process probably supported by cytokine signaling sustaining B-cell activation and proliferation.

In this context, some viral proteins like *core* protein, may directly modulate the mechanism underlying ICs deposition in the vascular bed leading to cryoglobulinemic vasculitis and promote proliferation signals of B cells supporting an active viral replication. In addition, host's genetic factors may represent a crucial factor for the clinical outcome of HCV chronic infection. These complex relations represent the biological basis for a more appropriate treatment of the cryoglobulinemic vasculitis that include antiviral therapy and B-cell depletion even if further studies are necessary for the relapsed-refractory cases in which other pathogenetic mechanisms, often antigen-independent, are involved.
